# Animal and Vegetable Physiology

**Published:** 1855-04

**Authors:** 


### *Animal and Vegetable Physiology.*

The nutritive functions of digestion, circulation, and assimilation, have for their object the support of life, by furnishing to organized beings, without ceasing, new and proper materials for the development of their organs, and the reparation of the waste occasioned by the movements of animality. But it is the nature of all organized beings to have but a limited existence. Their organs finally lose the faculty of sustaining life, and the cessation of their functions brings on death. All the organized beings existing on the earth, end by disappearing from its surface, and their races would become extinct if nature had not given them the means of reproducing and multiplying themselves.

Through reproduction the power of life is made to pass without ceasing into other bodies. Our parents are reproduced in us, as we are engendered anew in our descendants. Thus the indidual perishes, but the species is continued.

Wherever there is life there is attraction. The appearance of matter in organized beings implies a draught on the resources of nature. Each germinating seed exercises a special attraction on the earth and atmosphere, and dead inorganic matter collects around it to be embued with vitality, and moulded into an organized form. But there is only a limited amount of organizable material existing in nature, and her resources would therefore be speedily exhausted, were there not an equivalent amount returned. The matter which composes the bodies of animals and plants is only *borrowed* from the earth and atmosphere for a *little* time. A rotation of these substances is absolutely necessary.

Wherever there is death there is repulsion. The matter which was collected by life is scattered by death. The plant or animal decays and disappears from our sight. Both are alike dissolved by the repulsive principle into earthy elements and invisible gases, and the atoms held together by life thus sundered by death, once more roam through the universe, and gather round the living centres of attraction, to be again remouldered anew into living organized forms.

Matter, whether organized or inorganic, never perishes. Every atom bears on it the impress of its everlasting and infinite author. If it disappears from observation, it is only to enter into new combinations. You may crush the parts of a body to powder, melt it into a liquid, or, by a still intenser application of heat, dilate it into a gas, and dissipate it in vapor; but it still exists and can in many instances be collected again into the same body, without loss of weight or change of form. Mercury and water may be converted into vapor without the loss of a single particle.

The decay of animal and vegetable bodies, is only a process by which their particles are liberated to assume new forms.

What life borrows, death will sooner or later claim. The living incur a debt which must be paid. Matter is the grand circulating medium of nature, and all that is loaned—even to the minutest particle—must be returned. Nothing is ever lost by nature. Death is the agent employed to enforce the claim, and we must surrender what we have appropriated. We may be unwilling to pay the debt, but in this instance, at least, no fraud can be practised. We may cheat our fellow men out of "their own," but nature, never.

It is this ceaselsss return of organizable material which keeps up the continuity of the stream of life, and renders the fountain inexhaustible. Hence the matter of which every animal and vegetable was formed in the earliest ages, is still in existence. We, ourselves, are composed of matter as old as the creation; in time we must suffer, in our turn, decomposition, as every living body has done before us, and thus resign the matter of which we are composed to form new existences.

But it is not only by the dissolution of their fabric, but by the detachment of a portion of it at a certain period of time, in the form of an egg or seed, that organized beings reproduce each other. Each egg or seed encloses a miniature animal or plant called an embryo, which developes into the likeness of its parents, in consequence of receiving from them an influence which impresses on it its peculiar vital movements.

That all animals are produced from eggs, *omne vivum ex ovo*, is an old adage, abundantly confirmed by modern researches. In tracing back the successive phases of animal life, we invariably arrive at an epoch when the incipient animal was enclosed in an egg. It is then called an embryo, and the period passed in this condition, which is more or less long, is called the embryonic period.

Now, plants have sexes or sexual organs as well as animals. The female sexual organs in plants are called carpels. The pistil, or central organ of the flower, which consists of stigma, style and germen, is only a fully developed carpel. The male sexual organs are named stamens, the anthers of which contain the pollen or fecundating matter. The stamens and carpels are therefore the essential organs of reproduction in plants, and the parents of the vegetable embryo, since it is their natural action which contributes to its development.

The ovules contained in the germen are the bodies which, after impregnation, become seed. Their existence may be verified by making a section of the germen, whilst the flower is still in the bud, before the anther cells had been ruptured, and their polleniferous contents discharged. In this condition they undoubtedly correspond to the unimpregnated ova of animals. The line or ridge on the interior walls of the ovary, to which the ovules are attached, is called the placenta. The ovule is attached to the placenta, either directly, or by a prolongation or umbilical cord termed the funiculus. It is through this last organ that vessels pass into the ovule, which communicate with the nourishing walls of the germen, conveying to it those nutritive materials which contribute to its further development.

The reproductive function is exercised by animals and plants when they have attained to the full development of all their parts, or arrive at an adult state. The period when this occurs, varies greatly in each species, and depends entirely on the peculiarities of its constitution. When this epoch arrives in plants, a visible change in their outward appearance takes place ; the stem ceases to elongate, and its internodes no longer developing, the leaves remaining crowded together, in closely-approximated whorls, undergo peculiar modifications in form and coloring, and a flower is produced.

The period of ovulation is to animals what flowering is to plants, and indeed few phenomena are more interesting to the student of nature, than those exhibited by animals at the pairing season. Then their physiognomy is the most animated, their song the most melodious, and their attire the most brilliant. Some birds appear so different at this time that zoologists are always careful to indicate whether or not a bird is represented at the breeding season. Similar differences also occur among fishes and other animals whose colors are then much brighter than at any other time. Thus in early spring when plants put forth anew their leaves and flowers, we have renewed at the same time the feathers of birds, the hair of quadrupeds, the scales of fishes, &c. Both the animal and vegetable creation are alike re-clothed. It is the season of love and happiness. All nature rejoices. To many organized beings this season comes only once. In this respect many animals are like annuals amongst plants; the act of reproduction exhausts their vital energies, and they perish as soon as they have given birth to their eggs.

Reproduction, whether in animals or plants, is always attended with an exhaustion of their vital energies. The organized being either perishes at once in giving birth to its offspring, or the term of its existence is greatly abbreviated. Thus, as soon as the anther-cells or male sexual organs of flowers have discharged their pollen on the stigma of the pistil or female sexual organ, and the ovule in the germen has received the impregnating influence, the sap is immediately drawn from the other parts of the flower to the germen, which begins to swell and enlarge, and a series of changes soon announces that a new vitality is established in the impregnated parts to the detriment of the others. The flower, beautiful up to this moment, and adorned with the most lively colors, loses its pleasing aspect. The petals fade and fall. The stamens having fulfilled their functions, prove the same degradation. The germen alone remains in the centre of the flower,—the upper portion of it, the stigma and the style, is, however, useless to the plant, and, therefore, disappears equally with the other parts. The germen continues swelling and enlarges until it finally forms a fruit abounding with seed, by which the species is continued. It is not an unusual thing to see the calyx persistent with the germen, and contributing along with the green walls of the pericarp or seed vessel to the nourishment of the young embryo. The vitality of *all* the organs of the flower is, however, exhausted before the seed and the embryo which it contains are fully formed, and the calyx and pericarp alike perish when they have fulfilled this the last and most important function of vegetable life.

The seed or ovum is but a retreat into which exhausted vitality retires in order to recover its wonted energies; and in all the lower forms of organized being it is also a shelter for life during the prevalence of those conditions which are unfavorable for its development. Accordingly, we find that the seed of many early flowering annuals, germinates again in Autumn, as the light and heat of the sun are much the same as in early Spring. A little family of plants is thus seen rising around their aged and dying parent. In some instances the individuals of this family arrive again at an adult state, and flowers as well as leaves appear; generally, however, the germinating seeds can only produce leaves, the approach of cold weather arresting all further development. These and many other appearances in nature are deserving of a far greater share of attention than they have hitherto received. All practical gardeners and botanists are aware that there are many plants which flower in the Spring, and again develope in Autumn on the return of SIMILAR CONDITIONS of light, temperature, and moisture.

That the vegetable machinery would continue longer in motion, and simply stops in consequence of the decreasing light and heat of the sun, is evident from the fact that the annual and herbaceous plants of temperate climates become ligneous and persistent, in the warm and sunny regions of the tropics. The castor oil plant (*Ricinus communis*) for example, in Pennsylvania, puts forth large peltate-palmate leaves, and grows from three to eight feet in height, but is destroyed by the first frosts of Autumn. In the happy regions within the tropics its stem is ligneous and persistent, and it grows into a powerful and lofty tree. It is the same with the Euphorbiaceæ, Leguminosæ, Boraginaceæ, Labiatæ, Hypericaceæ, Verbenaceæ, Polygonaceæ, Rubiaceæ, Compositæ, and a host of other plants which we tread under our feet in Pennsylvania; but which elevate themselves majestically into the air in warm countries. Excepting on the mountain summit snow never falls on any other part of the landscape lying within the tropics, and the traveller wanders beneath the arborescent forms of Leguminosæ, of Boraginaceæ, and of Euphorbiaceæ, or if he be in the island of St. Helena, reposes beneath the shade of forests of Solidago of Sonchus, and of Echuim. The herbaceous and perishable annual has been transformed into the ligneous and enduring perennial. The plant whose humble growth and delicate beauty drew our admiration, as it grew at the foot of some oak or buttonwood, is now itself one of the noblest trees of the forest. Development has gone on, and we see the result of the magic influence of a continuity of solar warmth and brightness in the majestic form which now stands before our eyes. H. C.

*West Philadelphia, March* 13*th*, 1855.

